# Eastern equine encephalitis virus: Pathogenesis, immune response, and clinical manifestations

**DOI:** 10.1016/j.imj.2025.100167

**Published:** 2025-01-17

**Authors:** Bhumika Parashar, Rishabha Malviya, Sathvik Belagodu Sridhar, Tarun Wadhwa, Sirajunisa Talath, Javedh Shareef

**Affiliations:** a Department of Pharmacy, School of Medical and Allied Sciences, Galgotias University, Greater Noida 201310, Uttar Pradesh, India; b Galgotias Multi-Disciplinary Research & Development Cell (G-MRDC), Galgotias University, Greater Noida 201308, Uttar Pradesh, India; c RAK College of Pharmacy, RAK Medical & Health Sciences University, Ras Al Khaimah 11172, United Arab Emirates

**Keywords:** Neuroinflammation, Viral pathogenesis, *Alphavirus* transmission, Immune evasion, Neurological sequelae

## Abstract

Eastern equine encephalitis virus (EEEV) is a lethal *Alphavirus* transmitted by *Culiseta melanura* mosquitoes that primarily cycles between birds. Although rare, infections in humans and horses are associated with high mortality rates and severe neurological effects. Climate change appears to be increasing the spread of this virus. This study aims to provide a comprehensive analysis of EEEV, including its transmission dynamics, pathogenesis, induced host immune response, and long-term impacts on survivors. It also highlights the virus's unique immune evasion strategies that complicate disease management and contribute to severe clinical outcomes, such as encephalitis with fever, convulsions, and coma. Survivors often face chronic cognitive, motor, and psychosocial impairments. Despite these significant public health risks, gaps remain in understanding the molecular mechanisms underlying immune evasion and the long-term neurological sequelae in survivors. By collating current knowledge, this review underscores the urgent need for the development of targeted vaccines and therapeutic interventions to mitigate the growing threat of EEEV, particularly in the context of climate change-driven geographical expansion.

## Highlights

• Eastern equine encephalitis (EEE) virus spread by mosquito.
• Nearly 90 % of patients suffering from EEE virus lasting neurological sequelae, while the death rate for EEEV infections may reach 75 %.
• Severity of disease is also affected by environmental and climatic changes.
• The initial targets of virus infection are dendritic cells and fibroblasts, which cause the activation of self-defense systems.
• Patients may experience a loss of consciousness or coma, which ultimately leads to death.

## 1 Introduction

**Fig. 1:**
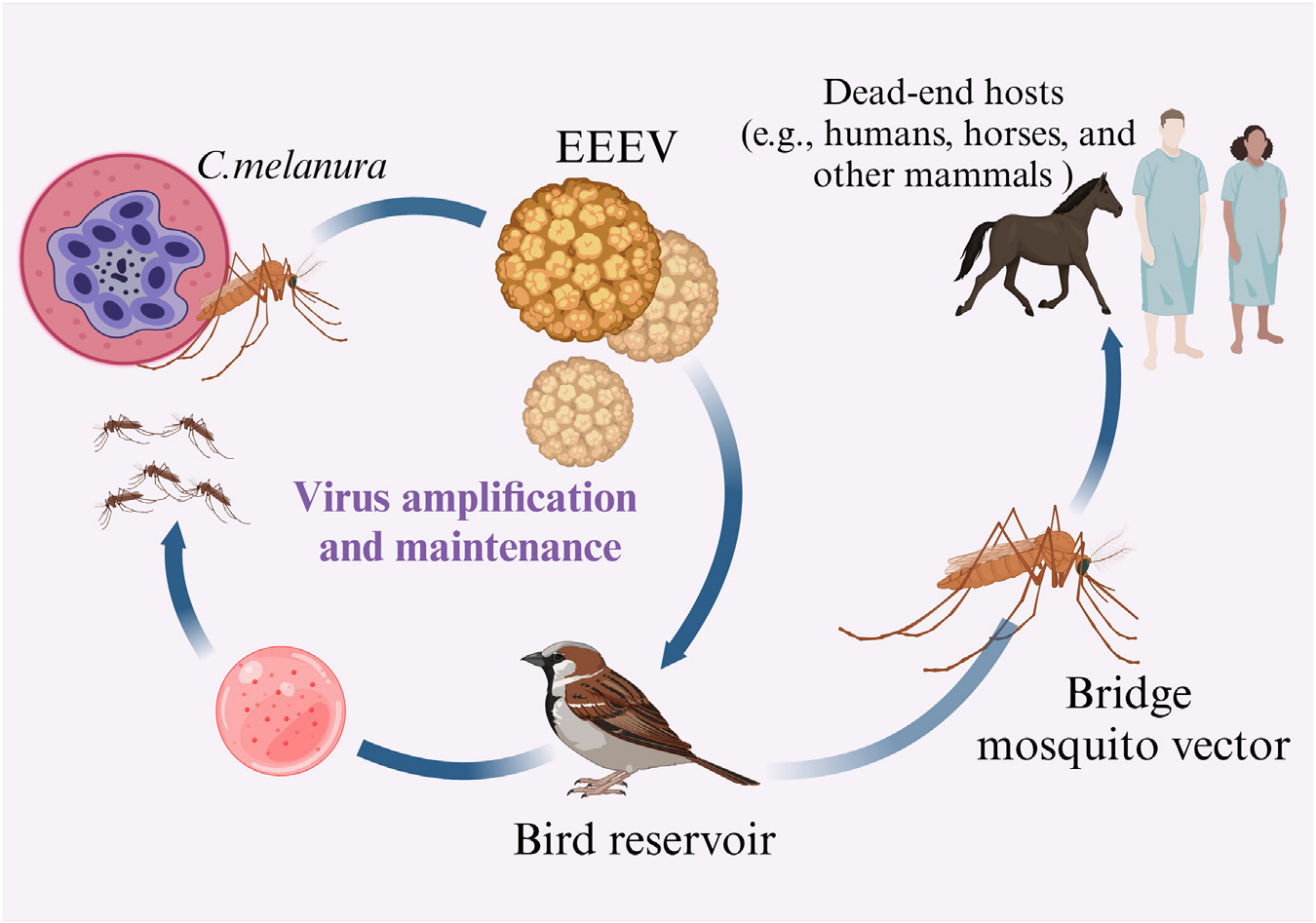
Transmission cycle of EEEV. The cycle involves the infection of *Culiseta melanura* mosquitoes while feeding on viremic birds, the maintenance of the virus within bird reservoirs, and the occasional spillover of the virus to humans and other mammals through bridging vectors. This cycle highlights the enzootic and epidemic dynamics of EEEV, underscoring the ecological and public health factors influencing its spread. *Abbreviations*: EEEV, eastern equine encephalitis virus.

Eastern equine encephalitis virus (EEEV) is the most severe *Alphavirus* impacting humans, with numerous survivors encountering neurological problems, including paralysis and cognitive impairment.^1^ Encephalitic alphaviruses include EEEV, western equine encephalitis virus (WEEV), and Venezuelan equine encephalitis virus (VEEV). EEEV is transmitted via the bite of an infected mosquito and encephalitis epidemics occur in equine species and humans.^2^ Transmission of the virus occurs most frequently in low-lying regions that are characterized by hardwood woodlands and marshlands, both of which are conducive to the growth of mosquito larvae.^3^ Avian species act as a reservoir for the virus and EEEV is primarily spread to birds via the *Culiseta melanura* mosquito. Environmental changes brought about by climate change could increase the risk of illness by affecting the population and distribution of the primary mosquito vector, thereby extending the season during which viruses are transmitted, and expanding the transmission reach to formerly uninhabitable areas.^4^ In humans, the mortality rate from EEEV infection can reach 75 %, with nearly 90 % of survivors suffering lasting neurological sequelae. The mechanisms by which EEEV infects and replicates in host cells have been investigated, particularly its neurotropic nature and the resulting damage to the central nervous system (CNS), including blood–brain barrier (BBB) disruption and neuronal inflammation.^5^ On occasion, the virus transmits from its typical reservoirs to infect terminal hosts, including humans, equids, pigs, pheasants, numerous game and exotic birds, and gallinaceous poultry. These spillovers transpire irregularly and are often facilitated by bridging vectors (non-enzootic mosquitoes that feed off avian and mammalian hosts), as seen in Fig. 1.^6^ From an epidemiological perspective, some horses exhibit elevated viremia levels, transferring the virus to feeding mosquitoes. This exacerbates the transmission of the virus in impacted regions. The innate and adaptive immune responses of the host to EEEV infection are being investigated, with a focus on how the virus evades immune detection and the implications for disease severity and outcomes.^7^ The illness known as encephalitis is characterized by inflammation of the brain parenchyma and is frequently induced by bacteria or viruses, in addition to being frequently linked to meningitis. Viruses exhibit considerable variability in their capacity to cause CNS infections. The extent of brain involvement and clinical outcome are influenced by the individual pathogen, the host immune status, environmental variables, the clinical symptoms and neurological complications associated with EEEV infection, as well as long-term sequelae faced by survivors, including cognitive and motor impairments. It is important to explore the virus's sophisticated mechanisms to evade host immune responses, including disruption of interferon (IFN) signaling pathways and pattern recognition receptor (PRR) engagement, which complicate disease management.^8^ There is currently an urgent need for effective surveillance, vaccines, and therapeutic interventions for EEEV, particularly in light of environmental changes that may lead to geographical expansion.^9^

## 2 Pathogenesis

EEEV belongs to the genus *Alphavirus*, within the family *Togaviridae*. EEEV is reported to infect neurons directly via a vascular pathway.^10^ Infected persons show acute manifestations including elevated temperature, cephalalgia, emesis, focal or widespread convulsions, and loss of consciousness. Additionally, there may be long-term neurological consequences.^11^^,^^12^ According to early molecular detection data, viral antigens inside osteoblasts, dendritic cells, and fibroblasts are crucial to pathogenesis.^13^ Skin proximal keratinocytes, ventricular interstitial cells, and ovarian stromal cells have also been identified as permissive cells.^14^ It is essential to investigate the specific roles played by non-structural proteins (nsPs) in modulating the host immune response to viral infection. The role of nsP1 is primarily in viral RNA capping, which is crucial for the stability and translation of viral RNA, and, by mimicking cellular mRNA capping, it helps the virus evade detection by the host immune system. By contrast, nsP2 plays a critical role in immune modulation. It acts as a protease and helicase, and, importantly, suppresses the host's IFN response. nsP2 can interfere with the host cell's nuclear import machinery, leading to the degradation of essential immune signaling molecules and transcriptional repression of interferon-stimulated genes (ISGs). In addition, nsP2 has been shown to inhibit the activation of key transcription factors such as STAT1, which are pivotal for initiating the antiviral state in cells. nsP3 is involved in disrupting cellular signaling pathways. It can interfere with the formation of stress granules, which are aggregates of proteins and RNAs that form in response to cellular stress and play a role in antiviral defense. By disrupting stress granule formation, nsP3 prevents the host cell from mounting an effective response to halt viral replication. Finally, nsP4 primarily functions as an RNA-dependent RNA polymerase and its activity is crucial for maintaining high levels of viral RNA, overwhelming the host cell's ability to respond effectively to infection. By targeting critical pathways involved in the detection and response to viral infection, nsPs play a central role in *Alphavirus* pathogenesis and immune evasion.^15^ Evidence has been reported of the presence of viral nucleic acid and antigen in cardiac and skeletal myocytes, as well as in the formation of teeth, epidermal epithelium, reproductive organs, and renal papillae in subsequent stages of infection, as shown in Table 1.

**Table 1:** EEEV transmission, pathogenesis, and clinical outcomes.

Category	Details
Virus name	Eastern Equine Encephalitis (EEE)
Family	Togaviridae
Genus	Alphavirus
Primary host	*Culiseta melanura* mosquito^16^
Transmission	Mosquitoes (*C. melanura*) feed on infected birds Humans and horses are infected through mosquito bites^17^
Replication process	The virus enters via mosquito bite Replicates in lymphoid tissues Spreads to the bloodstream Reaches the CNS causing encephalitis^18^
Primary host affected	Wild birds (asymptomatic carriers) Horses, pheasants, and humans (severe, often fatal symptoms)^19^
Transmission cycle	Enzootic cycle: Occurs during summertime in freshwater marshes, involving *C. melanura* mosquitoes Epizootic cycle: Occurs near marshes, and affects horses and humans^20^
Neurological impacts	Virus damages blood blood-brain barrier Causes edema, neuronal damage, and death Replicates in neurons and glial cells, leading to inflammation^21^
Symptoms in humans	Fever, headache nausea, vomiting, fatigue, muscle and joint pain Severe cases: Brain inflammation, seizures, paralysis, and death^22^
Host immune response	Stimulation of macrophages, dendritic cells, T lymphocytes, and B lymphocytes. The immune response facilitates inflammation, which leads to tissue and neural damage^23^
Pathogenesis	Virus replication in the brain. Inflammatory response exacerbates neural tissue damage. The involvement of microglia and astrocytes leads to further damage^24^
Outcome for survivors	Neurological complications or death in severe cases
Viral spread	Horses can contribute to virus spread when viremia levels are high enough for mosquitoes to transmit the virus^25^
Immune system activation	Stimulation of microglial and astrocytic cells The release of inflammatory mediators leads to further neural damage and cell death^26^
Therapeutic implications	Understanding the mechanisms of viral replication and immune response is critical for developing vaccines and treatments^27^

*Abbreviations*: EEE, eastern equine encephalitis; CNS, central nervous system.

### 2.1 Host immune response to EEEV

#### 2.1.1 Cytokines and the innate immune response

**Fig. 2:**
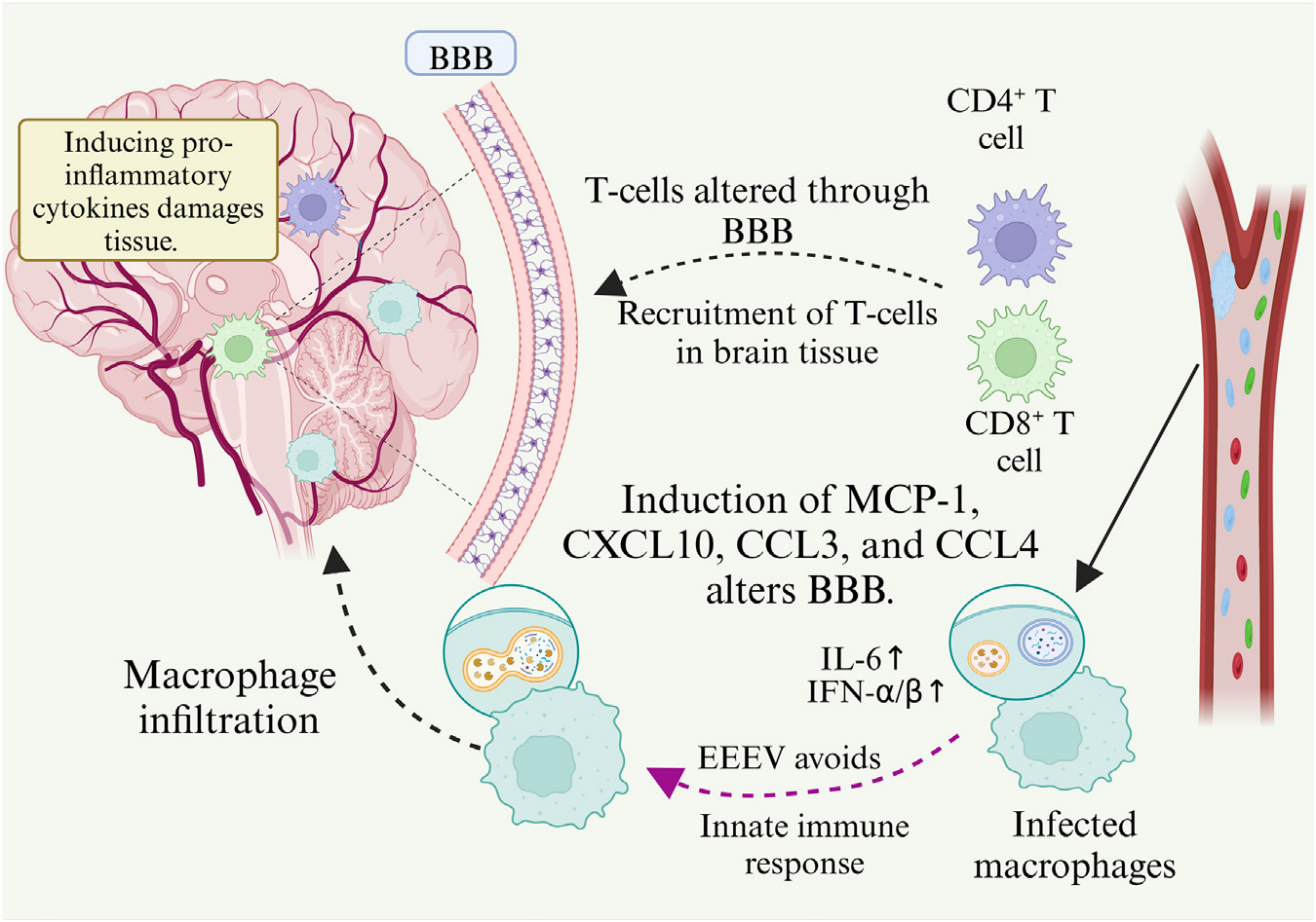
Infection with neurotropic EEEV is accompanied by the infiltration of T-cells and macrophages into the neurological system. This involves three steps: (1) Infection and evasion: EEEV infects macrophages, triggering cytokines (IL-6, IFN-α/β), while avoiding immune detection. (2) Blood–brain Barrier (BBB) disruption: chemokines (MCP-1, CXCL10, CCL3, CCL4) alter the BBB, allowing immune cell infiltration. (3) Central nervous system (CNS) inflammation: CD4^+^and CD8^+^ T-cells migrate to the brain, releasing pro-inflammatory cytokines, and worsening tissue damage. (4) Neurological impact: EEEV infects neurons, leading to encephalitis, CNS damage, and long-term impairments. *Abbreviations*: EEEV, eastern equine encephalitis virus; CNS, central nervous system; BBB, blood-brain barrier.

EEEV has developed strategies to circumvent the intrinsic immune response and to initiate infection.^28^ Innate and adaptive immune system reactions are elicited during the spread of viruses. PRRs are responsible for identifying pathogen-associated molecular patterns on viruses and initiating the production of IFNs throughout the body.^29^ Cells of the myeloid lineage, which include monocytes and macrophages, modify individual cytokine/chemokine profiles during infection with New World alphaviruses, facilitating viral dissemination within the host.^30^ A mouse study involving the spread of myeloid lineage innate immune cells using a replicon of VEEV that is insensitive to IFN and enclosed by structural proteins derived from EEEV, found that the effectiveness of infection differed between cells, as did the capacities of viral genomes to proliferate.^31^ EEEV shows suboptimal replication in lymphoid organs.^32^ In rodent models, EEEV demonstrates a tendency to spread to osteoblasts and mesenchymal fibroblasts.^33^ The penetration of myeloid cells, including lymphocytes such as monocytes and dendritic cells, into the internal organs is limited by the altered tropism of EEEV, which is linked to the evasion of IFN-α/β.^34^ Furthermore, EEEV invasion is limited by its interaction with heparan sulfate, which is a structural element of cell surface receptors that is essential for enhancing viral pathogenesis and neuronal infection, as well as viral transmission to the CNS.^34^^,^^35^ A factor reported to influence EEEV replication within myeloid cells is the microRNA, miR-142–3, found in myeloid cells that inhibits the replication of viruses by binding to the 3′ unaltered domain of EEEV and preventing viral progression.^36^ The inability of EEEV to enter innate cells means that robust IFN responses are avoided.^37^ EEEV attacks dendritic cells and macrophages within lymphatic tissues, along with Langerhans cells, additional dendritic cells, and skin macrophages.^38^ Within the brains of EEEV-infected mice, the amount of circulating monocyte chemoattractant protein-1 (MCP-1) is increased, which strongly affects the BBB, as shown in Fig. 2.^39^ The expression of MCP-1 is upregulated in the brain of EEEV infected mice and plays an important role in the direct alteration of the BBB.

#### 2.1.2 Adaptive immunity

Prior studies on animal models for EEEV indicated the need for several viral inoculations to elicit a T-cell helper immune response.^40^ Subsequently, it became apparent that innate as well as adaptive immune responses to EEEV are hindered when myeloid cells are unable to proliferate because of the virus.^41^ Widespread T-cell activation and differentiation, and the generation of IFN-α/β by myeloid cells, which ultimately results in the development of effector and memory T-cells, all play a vital role in the adaptive immune response.^42^ Alphaviruses can interfere with the antigen presentation process, which is crucial for the activation of adaptive immune responses. For example, nsP2 of Old World alphaviruses can induce global inhibition of transcription and translation in host cells, which includes the downregulation of major histocompatibility complex (MHC) molecules.^43^ This reduction in MHC expression hampers the ability of antigen-presenting cells to present viral antigens to T lymphocytes, thereby impairing the activation of CD8^+^ and CD4^+^ T-cells. Some alphaviruses have mechanisms to evade neutralizing antibodies, which are a key component of adaptive immunity. For example, the viral envelope proteins can undergo rapid mutation, allowing the virus to escape recognition by neutralizing antibodies. Additionally, the formation of stable cellular extensions or virus-induced filopodia can aid the spread of the virus between cells without being exposed to the extracellular environment and potential circulating antibodies.^44^

## 3 Clinical presentation

EEEV infection can manifest in a mild, self-resolving form or a severe, even lethal form with potentially serious neurological consequences in survivors.^45^ The prodromal stage is characterized by the presence of symptoms that are characteristic of a viral infection. These include fever and headache, which may be accompanied by lymphadenopathy, nausea, or vomiting.^46^ Indicators of CNS involvement, including alterations in mental state, heightened irritability and agitation, and shifts in personality, become apparent within a few days.^47^ Additionally, seizures, either focal or generalized, may take place. Localized neurological symptoms are occasionally seen.^48^ As a result, patients may experience a loss of consciousness or coma, which ultimately may lead to death. A stiff neck indicates involvement of the meninges. A fever is one of the most common symptoms, and a lack of fever raises questions regarding the diagnosis.^49^ In newborns less than 1-year-old and adults over 55 years, the prognosis is generally worse. In a study on significant cerebral edema, young children were particularly affected, as detailed in Table 2.^50^

**Table 2:** The clinical details of patients.

Aspect	Details
Symptoms onset	Symptoms started appearing anywhere from one to seven days before clinical presentation, with one day serving as the median; 1 patient had unknown timing^51^
Chief symptoms	Perplexity or changed state of mind: 3 patients Seizure: 2 patients^52^
Fever at presentation	6 patients reported fever; all were febrile within 24 h^53^
Premorbid conditions	There were no patients with preexisting neurologic impairments or main immunocompromising diseases^54^
Current treatments	No patients were undergoing immunomodulatory or anti-neoplastic treatment, and three of those individuals already had diabetes^55^
Initial physical examination	Five patients experienced altered levels of consciousness Three patients had seizures upon presentation^56^
Vital Signs	Febrile at presentation (≥ 38°C): 3 patients Tachycardia (pulse > 100 bpm): 6 patients Tachypnea (respiration ≥ 20 bpm): 5 patients^57^
Neurologic findings	6 localized abnormalities (facial droop, myoclonic jerks), aphasia, and amnesia were observed in five patients with aberrant neurologic findings^58^
Admitting diagnoses	Acute encephalopathy and stroke were the most common, each affecting 2 individuals; meningoencephalitis, aseptic meningitis, community-acquired pneumonia, and undifferentiated sepsis were among the other diagnoses^59^

### 3.1 Long-term sequelae

EEEV may have serious long-term consequences, such as cognitive impairment, movement deficiencies, and other neurological problems. Studies indicate that while few individuals may fully recover, many experience long-term sequelae that can profoundly affect their quality of life.^60^

#### 3.1.1 Neurological impairments

##### 3.1.1.1 Neuroinvasive disease

EEEV infection can lead to severe neuroinvasive disease, characterized by symptoms such as headache, meningism, confusion, focal neurologic deficits, seizures, and coma.

##### 3.1.1.2 Cerebrospinal fluid (CSF) findings

CSF analysis often shows an initial neutrophil-predominant pleocytosis that shifts to lymphocyte-predominance, along with elevated protein levels and normal glucose levels.

##### 3.1.1.3 Neuroimaging

Neuroimaging, particularly MRI, reveals characteristic lesions consistent with encephalitis, including neuronal destruction and vasculitis in the cortex, midbrain, and brain stem. T2-weighted images often show areas of increased signal in the basal ganglia and thalami, which is a distinctive feature of EEEV infection.

##### 3.1.1.4 Cognitive dysfunction

Memory, attention, and executive function issues are common among survivors. Cognitive impairments may vary in severity, from little memory loss to severe problems that substantially affect day-to-day functioning.^61^

##### 3.1.1.5 Motor deficits

Patients may have problems with coordination, spasticity, or weakness. These motor deficits might make it harder to maintain balance or carry out fine motor activities.^62^

##### 3.1.1.6 Seizures

Certain patients acquire seizure disorders post-EEEV infection, requiring continuous therapy with antiepileptic drugs.^63^

#### 3.1.2 Psychological effects

##### 3.1.2.1 Mood disorders

The likelihood of individuals who survive suffering from post-traumatic stress disorder, depression, and anxiety is higher than that of healthy individuals. Because of the severity of the disease and the repercussions of the illness, these problems can become even more severe.^64^

##### 3.1.2.2 Behavioral alterations

The patient may display modifications in personality or conduct, potentially straining relationships and complicating social reintegration.^65^

#### 3.1.3 Physical health issues

##### 3.1.3.1 Fatigue

Chronic exhaustion is often reported, reducing the patient's capacity to participate in routine activities or employment.^66^

##### 3.1.3.2 Pain syndrome

Patients, especially those with problems with their nervous system or muscles and joints, may live with persistent pain or discomfort.^67^

#### 3.1.4 Rehabilitation

##### 3.1.4.1 Rehabilitation therapies

Physical therapy, occupational therapy, and speech therapy are typically necessary for survivors to address motor skills, communication difficulties, and daily living activities.^68^

##### 3.1.4.2 Multidisciplinary approach

Long-term sequelae are often managed by a multidisciplinary team that includes neurologists, psychiatrists, physical therapists, and rehabilitation experts.^69^

### 3.2 Laboratory and clinical characteristics

If the level of functioning of a patient after being released from an urgent care facility is classified as normal, this indicates the absence of any remaining deficits.^70^ By contrast, a diagnosis of substantial sequelae means that a patient may require treatment in an institution, whilst a diagnosis of light sequelae suggests that self-care may be accomplished outside of an institutional setting. It may be necessary to assist a patient with daily activities if they are described as having substantial sequelae, which can indicate that they are in a continuous vegetative state. Other patients do not survive the disease and die whilst in hospital.^70^, ^71^, ^72^ Table 3 provides a comprehensive analysis of the clinical and laboratory characteristics of a cohort of patients who were diagnosed with EEEV infection. Particular attention was paid to mortality, neurological symptoms, laboratory results, and overall patient outcomes.^73^

**Table 3:** The clinical and laboratory characteristics of EEEV-infected patients.

Characteristic	Details
Mortality rate	13 of 36 patients died (36 %)^74^
Survivor outcomes	1 individual has entirely recovered 14 exhibited modest limitations 3 exhibited moderate limitations 5 exhibited significant deficits^75^
Duration of hospitalization	18 days (interval: 1–89 days)^75^
Neurological status at admission	4 patients were stuporous or comatose upon evaluation 21 others became stuporous within 2 days (a total of 69 %)^76^
Overall stupor/Coma rate	32 of 36 patients (89 %) exhibited stupor or coma^77^
Duration of coma (favorable outcomes)	5 days (standard deviation: 4.7 days) 1 patient recovered with modest sequelae following 9 days of unconsciousness^78^
Convulsions	Occurred in 18 patients: 15 generalized (most tonic-clonic, 2 twitching) 3 focal (1 also had generalized) 1 partial complex^79^
Focal weakness	Present in 16 patients (spanning from slight to severe), paresthesias accompanied by weakness in 1 patient^80^
Cranial nerve paralyses	Formulated in 9 patients: Cranial nerve VII in 5 Cranial nerve XII and oculomotor symptoms in 5 instances^81^
Clinical course	1 patient course indistinguishable from aseptic meningitis^82^
Leukocytosis	Median white cell counts at admission: 14 500 cells/mm³ (range: 3 800–23 900 cells/mm³)^83^
Hyponatremia	Median serum sodium concentration: 134 mmol/L (range: 123–146 mmol/L)^84^
Lumbar puncture	34 individuals (94 %) underwent at least one lumbar puncture^84^
Initial CSF findings	Median leukocyte count: 370 cells/mm³ (range: 0–2 400 cells/mm³; median 70 % neutrophils) Median protein: 97 mg/dL (range: 31–297 mg/dL) In most cases, hypoglycorrhachia is not present^85^
Encephalography findings	Accessible for 24 patients: All of them displayed a generalized slowing down and disarray of the backdrop elements Discharges of an epileptic nature in 6 patients (4 with periodic lateralizing discharges) No specific correlations with clinical events^86^
Prodrome duration	This condition frequently mimics benign viral sickness (fever, headache, gastrointestinal distress), with a median duration of 5 days and a range of 0 to 28 days^87^
Neurological symptoms	Symptoms prompted evaluation: convulsions, meningeal signs, confusion, and somnolence are some of the symptoms that may be present^88^

*Abbreviations*: CSF, cerebrospinal fluid.

## 4 Immune evasion

The immune evasion methods of alphaviruses are essential to their pathogenesis.^89^ The control of viral replication by the host intrinsic immune response is profoundly impacted by essential viral RNA structures.^90^ The elimination of viral infection relies on both the innate and adaptive immune responses of the host, and differences in the host immunological response impact the intensity of the disease.^91^ Alphaviruses have developed many strategies to inhibit host reactions, including premature evasion techniques to increase permissiveness within the diseased host.^92^ Viral evasion strategies often target type I IFN, owing to its pivotal function in initiating intrinsic and adaptive immune system reactions, which are crucial in managing pathogenic viruses.^93^ Viruses affect every step of the signaling pathways involving IFN. The cyclic-GMP-AMP synthase (cGAS) - stimulator -STING (stimulator of interferon genes) signaling pathway is crucial for initiating IFN gene production in response to tissue injury, cellular stress, and infection.^94^ The transcription and expression of the cGAS-STING innate immune pathway could inhibit virus replication, whereas viruses could antagonize this process. Chikungunya virus (CHIKV) infection markedly reduces cGAS expression, although STING expression remains constant.^95^ The interaction between nsP1, a viral non-structural protein, and STING affects the generation of IFNs and the induction of IFN-β promoter activity, which is typically stimulated by the cGAS-STING pathway.^96^ Toll-like receptors (TLRs), retinoic acid-inducible gene (RIG), and nucleotide oligomerization domain (NOD) are examples of host PRRs, which are responsible for triggering signaling cascades that ultimately lead to the production of type I IFN.^97^ NOD found in skin and innate immune cells sense viral components and play a critical role in the production of cytokines.

ISGs are essential for regulating the replication of Ross River virus, Sindbis virus (SINV), and O'nyong-nyong virus.^98^ Host PRRs such as RIG, NOD, and TLRs allow alphaviruses to adhere to and enter the host, and induce type I IFNs. When alphaviruses infect host cells, cytokines disrupt the type I IFN pathway in numerous ways, including blocking the cGAS-STING signaling pathway.^99^ In addition to SINV nsP1 and CHIKV nsP2, proteins E1/E2 are also capable of inhibiting activation of the IFN-β promoter, which affects the MDA5/RIG-I receptor signaling pathway.^100^^,^^101^ The nsP2s of CHIKV, Mayaro virus, SINV, and Semliki Forest virus were found to influence ISGs by altering palmitoylation and phosphorylation, thereby hindering the production of IFN-α/β and ISGs. Lastly, CHIKV impairs the phosphorylation of STAT1, which is essential for the IFN-induced JAK-STAT pathway to produce viral RNA inhibitors that circumvent RNAi-based defense systems in eukaryotes, as demonstrated by the Semliki Forest virus.^102^ Alphaviruses, such as CHIKV, employ strategies to avoid detection by the adaptive immune response throughout their life cycle.^103^ These strategies include the development of long-lasting cellular extensions that shield the virus from neutralizing antibodies and facilitate viral transmission between cells.^104^

## 5 Nervous system complications

**Fig. 3:**
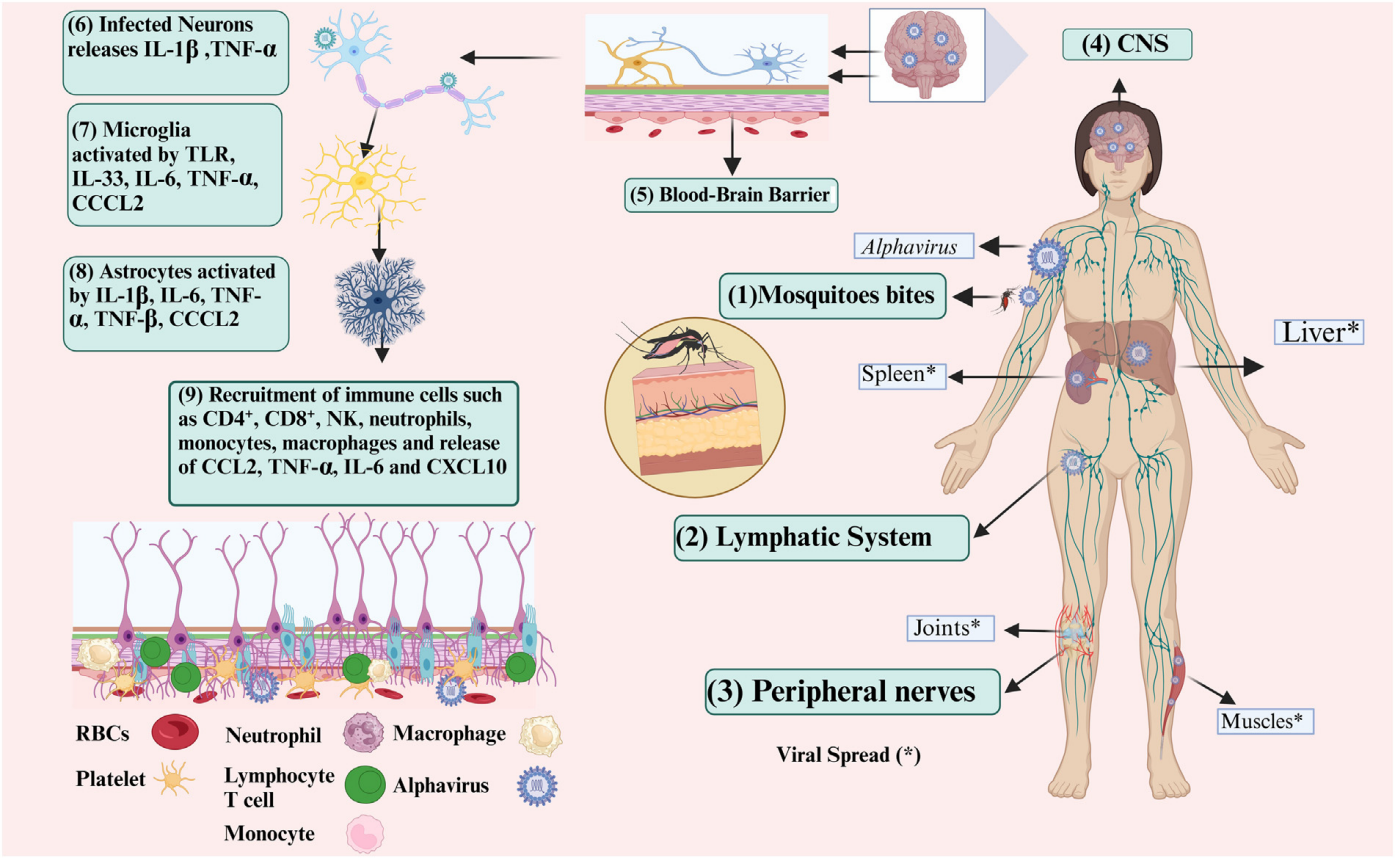
Proposed mechanism of EEEV pathogenesis from skin entry to neuroinflammation. The mechanism involves the following steps: (1) Virus-infected mosquitoes breach the epidermal barrier by injecting saliva. (2) The virus employs pattern recognition receptors, including toll-like receptors (TLRs) and RIG-I-like receptors (RLRs), (3) to activate the innate immune responses of dendritic cells and fibroblasts. (4) The immune response enlists macrophages and immune cells, leading to the synthesis of pro-inflammatory cytokines (IL-6, TNF-α), chemokines (CCL2, CXCL10), and interferons. (5) The virus infiltrates adjacent lymph nodes via dendritic cells, where it proliferates before entering the bloodstream and causing viremia. (6) The virus negatively impacts the kidneys, muscles, joints, spleen, and, in severe cases, the brain. The virus penetrates the brain via the peripheral nerves and circulation, entering the central nervous system (CNS). (7) Infected neurons release signals, such as TNF-α and IL-1β, which activate glial cells. Microglia, specialized macrophages acting as sentinel cells, are the primary responders to neuronal infection by recognizing danger signals from neurons through receptors such as Toll-like receptors. (8) The activation of microglia subsequently triggers the activation of astrocytes. (9) Astrocytes recruit immune cells, including CD4^+^ T-cells, CD8^+^ T-cells, natural killer cells, neutrophils, monocytes, and macrophages, to the infection site and release chemokines and cytokines such as CXCL10, CCL2, TNF-α, and IL-6, which promote neuroinflammation and modify blood–brain barrier permeability. *Abbreviations*: EEEV, eastern equine encephalitis virus; TLRs, toll-like receptors; RLRs, RIG-I-like receptors; CNS, central nervous system.

EEEV is associated with the development of neurological disorders.^105^ An increase in neurological issues has been observed in association with viruses not commonly linked to these complications. CHIKV is an *Alphavirus* that originated in the Old World and was primarily associated with joint discomfort but has also been associated with unusual and serious neurological problems such as meningoencephalitis and encephalopathy,^106^ as summarized in Fig. 3. Among its unusual presentations, the neurological problems caused by CHIKV infection have the highest recurrence and fatality frequencies.^107^ Patients with CHIKV-induced neurological difficulties demonstrate increased cytokine (IL-6, IFN-α, TNF-α) and chemokine (CXCL9, CCL2, CCL5, CCL7) concentrations in their CSF compared with individuals with no neurological symptoms.^108^ CCL2 is essential for the recruitment of monocytes to the brain, particularly the cluster of cells that express CD14 and CD16, which may play a Trojan horse function facilitating CHIKV entrance into the CNS.^109^ Two pathways suggested to be involved in CNS complications associated with CHIKV include direct CNS contagion via a detrimental inflammatory response and choroid plexus. This was validated by evidence indicating alterations in the PECAM-1 concentration in infected cells. Furthermore, disruption to endothelial cells influencing permeability, the BBB, and metabolomic and proteomic patterns suggest immunological disruption, inflammation, and damage to the endothelium, accompanied by changes in protein levels. Excessive inflammation in severe infections may result in endothelial damage, deregulation of the anticoagulation cascade, and diminished vasopressin levels, hence contributing to hemodynamic abnormalities.^110^ Mosquitoes harboring the virus, use their proboscis to inject saliva that contains the virus into human skin, breaking through the skin's protective layer. The initial targets of the virus are dendritic cells and fibroblasts, which cause activation of self-defense systems through recognition by PRRs,^111^ including RIG-I-like receptors (RLRs) (e.g., RIG-I and MDA5) that recognize viral double-stranded RNA, initiating a signaling cascade through mitochondrial antiviral-signaling protein (MAVS),^112^ leading to type I IFN production. Type I IFNs are critical for mounting an effective antiviral response by activating the JAK-STAT pathway, which leads to the expression of ISGs.^113^ These ISGs restrict viral replication and spread. However, EEEV nsPs, particularly nsP2, inhibit IFN signaling by degrading key components of the JAK-STAT pathway, such as STAT1 and STAT2. This blocks ISG activation, promoting viral persistence. The failure to resolve infection allows the virus to reach the CNS, where it causes severe inflammation, neuronal death, and breakdown of the BBB, and TLRs, TLR3 and TLR7, detect viral RNA in endosomes, triggering a similar IFN response through adaptor proteins, such as TRIF, or a myeloid differentiation primary response.^88^ However, EEEV nsP2 disrupts this signaling by inhibiting the nuclear translocation of transcription factors, such as IRF3 and NF-κB, which are essential for IFN production. This suppression prevents the activation of antiviral effector genes, allowing the virus to replicate unchecked. This immune evasion contributes to higher viral loads, which correlate with more severe disease outcomes, particularly neuroinflammation and encephalitis. The inhibition of PRR and IFN pathways delays the host's ability to mount an immune response, allowing EEEV to replicate to high levels in peripheral tissues and spread to the CNS. This unchecked viral replication and immune evasion facilitate neuroinvasion. In the CNS, the virus induces a hyperinflammatory response, leading to severe encephalitis. This manifests as neuronal damage, seizures, and high mortality rates. The virus's interference with PRRs and IFN signaling creates an imbalance in the immune response, leading to excessive production of pro-inflammatory cytokines (e.g., IL-6, TNF-α). This contributes to tissue damage in the brain, compounding disease severity.^114^ At the beginning of this process, a chain of immunological responses is triggered, involving the influx of monocytes as well as additional immune cells, along with the release of cytokines, such as TNF-α and IL-6, IFNs (IFN-β and IFN-α), and chemokines (CCL2 and CXCL10), which induce inflammation.^115^

Tolerance intends to limit pathogen spread to adjacent areas through the body, utilizing the lymph nodes and migratory immune cells, particularly dendritic cells. Tolerance intends to limit pathogen spread to adjacent areas through the body, utilizing the lymph nodes and migratory immune cells, particularly dendritic cells.^116^ Lymph nodes are the sites of intensive viral multiplication prior to the viral progeny accessing the circulatory system, leading to viremia.^117^ The infection can spread to organs, such as the liver and spleen, to muscles and joints, and even to the brain in extreme cases.^118^ The brain is responsible for transmitting data via nerves that exist in the peripheral and circulatory system. Upon entering the CNS, the infection undermines the integrity of the BBB through methods including Trojan horse transcytosis and direct disruption of type I IFN signaling, which plays a crucial role in maintaining BBB integrity during *Alphavirus* infection.^119^ IFNs can differentially restrict the transcytosis process at the BBB and inhibit virus replication within brain microvascular endothelial cells (BMECs) and pericytes.^120^ This signaling pathway helps in localizing tight junction proteins at the cell borders of BMECs, thus maintaining the barrier's integrity. Besides transcytosis, alphaviruses can also use a Trojan horse mechanism to enter the CNS.^121^ Infected leukocytes, such as macrophages and dendritic cells, can act as carriers for the virus, introducing viral particles into the CNS upon their infiltration. This mechanism involves the interaction and extravasation of leukocytes at the BBB, facilitated by the expression of cell adhesion molecules and chemokines. The infiltration of these immune cells can lead to an increase in viral load within the CNS.^122^ Infectious synapses emit impulses, such as IL-1 and TNF-α, which activate cells of the glial tissue. Microglia, which are specialized monocytes of the CNS, operate as sensor lymphocytes and serve as primary responders to neural inflammation, identifying warning messages from synapses via receptors such as TLRs.^123^ Stimulation of the glial occurs after the activation of microglia. Activated astrocytes secrete several cytokines, including IFN-γ, TNF-α, IL-6, IL-1β, TGF-β, and CCL2, which facilitate neurological inflammation and alter the BBB permeability.^124^ Additionally, these astrocytes elicit an inflammatory response by attracting immune lymphocytes, such as CD4^+^ T-cells, CD8^+^ T-cells, natural killer cells, neutrophils, monocytes, and macrophages, while secreting certain cytokines (TNF-α, IL-6) and chemokines (CXCL10, CCL2).^125^

## 6 Conclusions

EEEV is a highly pathogenic arbovirus that primarily spreads via mosquitoes, causing severe encephalitis in humans and animals. EEEV is mostly transmitted by the *C. melanura* mosquito and cycles between birds and mosquitoes, with humans and horses serving as terminal hosts. Although rare, human infections are life-threatening, with mortality rates reaching up to 75 %, and survivors often face significant neurological impairments. The pathogenesis involves viral replication in the brain, causing inflammation, destruction of the BBB, and extensive neuronal damage. EEEV elicits both innate and adaptive immune responses, but the virus has evolved mechanisms to evade the host immune system, which worsens disease outcomes. Alphaviruses can lead to significant human diseases, with consequences that vary from chronic arthritis to potentially fatal encephalitis. The hostility of mosquito vectors and their proximity to host species, in addition to the possibility of aerosol transmission for certain alphaviruses, have culminated in the classification of some of these viruses as possible biological weapons by authorities. Numerous factors that contribute to the success of these viruses as pathogens have emerged over time and include their wide cell tropism, anti-IFN mechanisms, control of cytokine and chemokine responses, and adaptability to animal vectors that reside in urban areas. Climate change increases the risk and spread of EEEV, as the environmental shifts create favorable conditions for mosquito vectors. The long-term consequences of EEEV infection in survivors include cognitive deficits, motor dysfunction, and psychological issues. Rehabilitation is often needed for survivors, who may suffer from permanent disabilities. EEEV is a growing concern because of its severe health impacts and the potential for expanded geographical spread, raising the public health risk, particularly in regions where mosquito populations thrive. Efforts to control the spread of the virus and develop vaccines or treatments are critical in mitigating the public health risks posed by EEEV. Future research should focus on understanding the molecular mechanisms of immune evasion and identifying targets for therapeutic intervention. This will aid the development of strategies to mitigate the growing threat of EEEV, particularly in regions of high mosquito abundance.

## Funding

None.

## CRediT authorship contribution statement

**Bhumika Parashar:** Writing – original draft, Data curation. **Rishabha Malviya:** Writing – review & editing, Supervision. **Sathvik Belagodu Sridhar:** Data curation, Conceptualization. **Tarun Wadhwa:** Methodology, Data curation. **Sirajunisa Talath:** Supervision, Resources, Project administration. **Javedh Shareef:** Resources, Data curation.
